# Surface‐Guided Radiotherapy for Vulvar Cancer in the Frog‐Leg Position: Setup Accuracy and BMI Effects

**DOI:** 10.1002/acm2.70714

**Published:** 2026-07-26

**Authors:** Hao Liang, Junfang Yan, Yongguang Liang, Xiansong Sun, Hongming Li, Huiying Qu, Yijun Wang, Jingyu Lin

**Affiliations:** ^1^ Department of Radiation Oncology Peking Union Medical College Hospital, Chinese Academy of Medical Sciences and Peking Union Medical College Beijing China

**Keywords:** Body Mass Index (BMI), Frog‐leg Position, Setup Errors, Surface‐Guided Radiotherapy (SGRT), Vulvar Neoplasms

## Abstract

**Background:**

Radiotherapy for vulvar carcinoma often employs the “frog‐leg” position to minimize skin toxicity, but this position is biomechanically unstable and prone to setup errors using conventional skin markings.

**Purpose:**

To evaluate the clinical efficacy of Surface‐Guided Radiotherapy (SGRT) for setup accuracy in vulvar cancer patients treated in the frog‐leg position and to quantify the biomechanical correlation between Body Mass Index (BMI) and setup errors.

**Methods:**

A retrospective cohort study of 20 vulvar cancer patients treated in the frog‐leg position was conducted. All patients were treated using a uRT‐linac 506c accelerator with integrated helical Fan‐Beam CT (FBCT). Daily image guidance was performed using a two‐step registration protocol (bony anatomy followed by soft‐tissue fine‐tuning). Setup errors from 500 FBCT datasets were compared between the conventional laser group (n = 10) and SGRT group (n = 10). The correlation between BMI and setup errors was analyzed using Spearman's rank correlation.

**Results:**

The SGRT group demonstrated significantly superior setup accuracy across multiple key dimensions (*p* < 0.001). Notably, the Pitch error was reduced from 1.21° ± 0.48° in the Conventional Group to 0.07° ± 0.32° in the SGRT Group (Cohen's *d* = 2.79). Longitudinal (LNG) error decreased from 0.49 ± 0.26 cm to 0.04 ± 0.24 cm (*d* = 1.82), and vertical (VRT) errors were similarly reduced.

**BMI correlation:**

In the Conventional Group, BMI showed a strong significant correlation with Pitch and Yaw errors (*ρ* = −0.81, *P *= 0.005), indicating that obesity may exacerbate pelvic rotational instability. Conversely, in the SGRT Group, no statistically significant correlation was observed between BMI and setup errors in any dimension (*p* > 0.05), demonstrating the system's robustness against body habitus variations.

**Conclusion:**

The frog‐leg position is characterized by significant biomechanical instability due to distinct anatomical factors. Traditional skin marking methods are susceptible to the “skin traction effect” and interference from BMI. SGRT, through real‐time topographical matching, effectively corrects pelvic tilt and longitudinal sliding. Crucially, it eliminates the setup uncertainty associated with BMI, providing a solid evidence‐based foundation for precision radiotherapy and individualized PTV margin reduction.

## 1 INTRODUCTION

Vulvar carcinoma represents approximately 4%–5% of malignancies in the female reproductive system^1^, ^2^ For patients with locally advanced disease or those ineligible for surgical resection, concurrent chemoradiotherapy remains the standard of care^3^ Due to the deep anatomical folds in the inguinal region, radiotherapy is prone to the “bolus effect,” which can induce severe radiation dermatitis and compromise dose homogeneity within the target volume. To mitigate this clinical challenge, the “frog‐leg” position—characterized by flexion and abduction of the lower extremities—is routinely adopted to adequately expose the inguinal region and optimize dose distribution^4^

While the frog‐leg position offers distinct dosimetric and skin‐sparing advantages, it introduces significant challenges to setup accuracy due to inherent biomechanical instability^4^ Conventional setup workflows rely heavily on the alignment of room lasers with skin marks (tattoos) on the patient's surface. However, evidence suggests that Body Mass Index (BMI) is a critical independent predictor of setup errors in pelvic radiotherapy^5^, ^6^, ^7^ The soft tissues of the vulva and lower abdomen are highly mobile; influenced by BMI and organ filling, the relative displacement between surface markers and internal bony structures (i.e., skin‐to‐bone displacement) is often substantial, with error magnitudes potentially reaching 10–20 mm^8^, ^9^

For many patients with a high BMI, the thickened subcutaneous adipose layer not only may exacerbate soft tissue deformation but also induces a “skin traction effect” during frog‐leg positioning, rendering the traditional “single‐point alignment” mode potentially ineffective. This is particularly problematic for pelvic tilt (Pitch); as lateral skin marks are often situated near the axis of rotation, subtle deep skeletal rotations are imperceptible to the naked eye. This undetected rotation may lead to under‐dosing of target margins—specifically the inguinal lymph nodes—or excessive dose delivery to organs at risk (OARs)^10^

Surface‐Guided Radiotherapy (SGRT), an emerging auxiliary localization technology, utilizes optical cameras to capture the patient's three‐dimensional surface topology, providing real‐time positional feedback with submillimeter accuracy. The AAPM Task Group Report 302 explicitly highlights the immense potential of SGRT in enhancing setup precision and facilitating mark‐less radiotherapy^11^, ^12^, ^13^Although SGRT has been proven to significantly improve setup accuracy in breast and pelvic tumors^14^ quantitative research regarding its application in the anatomically complex and biomechanically unstable frog‐leg position—particularly concerning the correction of BMI‐related errors—remains limited in contrast to the extensive literature available on standard supine positioning.

This study aims to quantify the efficacy of SGRT versus conventional skin marking in controlling six‐dimensional (6D) errors in the frog‐leg position through a retrospective cohort analysis. Furthermore, we specifically explore the intrinsic relationship between BMI and axial errors, providing evidence for individualized margin strategies in precise vulvar cancer radiotherapy.

## 2 MATERIALS AND METHODS

### 2.1 Patient selection

This retrospective cohort study was approved by the Ethics Committee of Peking Union Medical College Hospital (Ethics No. JS‐3530) and registered on ClinicalTrials.gov (NCT05853549). Inclusion criteria were: (1) histopathologically confirmed squamous cell carcinoma of the vulva; (2) receipt of definitive or adjuvant Intensity‐Modulated Radiation Therapy (IMRT) or Volumetric Modulated Arc Therapy (VMAT); (3) standardized treatment in the frog‐leg position; and (4) availability of complete daily IGRT imaging data.

A total of 20 patients were enrolled and stratified into two groups based on the setup method: All patients in this study were treated using the uRT‐linac 506c (United Imaging Healthcare, Shanghai, China). This system is uniquely characterized by its integrated diagnostic‐grade helical Fan‐Beam CT (FBCT) on the same gantry, providing superior soft‐tissue contrast for daily image guidance compared to conventional CBCT^15^All patients in this study were treated using the same uRT‐linac 506c and the same AlignRT optical system (Vision RT, UK) to eliminate hardware‐induced variability.

Conventional Group (*n* = 10): Utilized a standard workflow based on skin markings and a three‐point laser system.

SGRT Group (*n* = 10): Utilized the optical surface monitoring system for 6D positional verification following initial laser alignment.

### 2.2 Simulation and planning

All patients were immobilized using a customized foam vacuum cushion, encompassing the lower body from the waist down, including both lower extremities and feet. Patients were positioned supine with knees flexed and abducted, and feet placed sole‐to‐sole to fully expose the inguinal region. Simulation was performed using a large‐bore CT (Spectral, Philips, Netherlands) with a 5 mm slice thickness. Target volumes and OARs were delineated using the uTPS treatment planning system (Version R001.3.0, United Imaging Healthcare, Shanghai, China). For the SGRT Group, upon plan completion, the external body contour structure was transferred to the AlignRT optical system (Vision RT, UK) to serve as the Reference Surface for subsequent setups^11^

### 2.3 Setup procedures

To minimize interobserver bias, all positioning procedures were performed by a consistent team of senior radiation therapists. Initial coarse positioning was performed using lasers. The SGRT system underwent daily quality assurance (QA) and calibration checks using a dedicated phantom to ensure submillimeter and sub‐degree accuracy throughout the study period.

Conventional Group: Therapists aligned the patient by matching the room lasers to cross‐marks drawn on the patient's abdomen and lateral pelvis during simulation, adjusting the couch and patient posture until coincidence was achieved.

**FIGURE 1:**
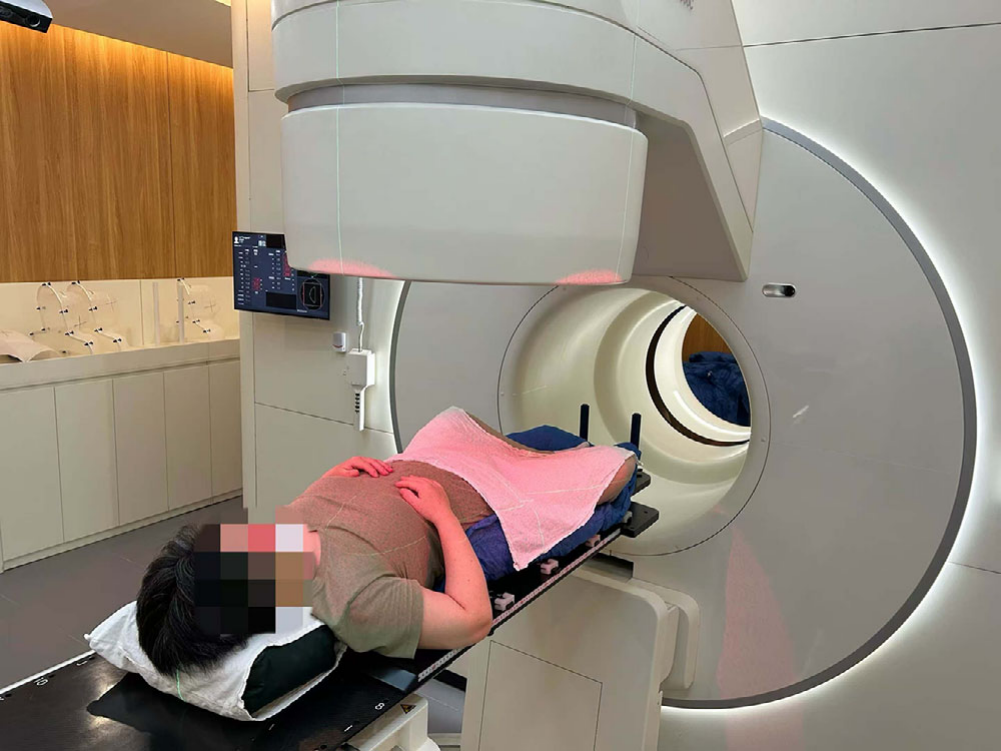
Clinical setup and surface guidance.

**FIGURE 2:**
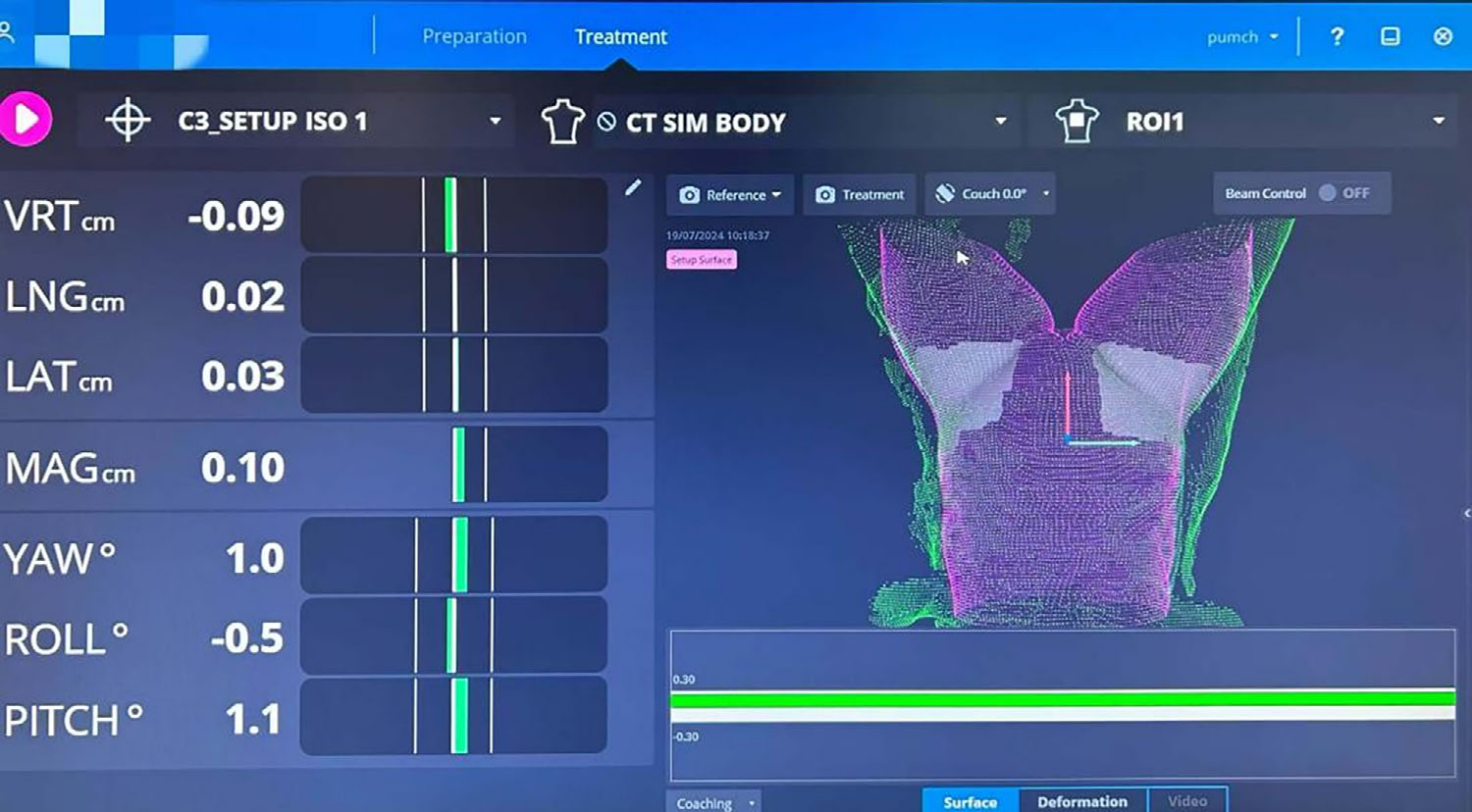
The SGRT system interface (AlignRT) displaying the Region of Interest (ROI) selection (pink/red overlay) on the pelvic surface. The real‐time deltas values for six degrees of freedom are monitored to ensure setup accuracy within the tolerance threshold.

SGRT Group: Initial coarse positioning was performed using lasers. The SGRT system was then activated, and a region of interest (ROI) was selected. The ROI encompassed the superior surface of the pelvis and proximal thighs, with the upper abdomen strictly excluded to avoid respiratory motion interference, as well as the edges of the immobilization device. The system displayed real‐time 6‐dimensional (6D) positional deviations (translations: ΔLat, ΔLng, ΔVrt; rotations: ΔPitch, ΔRoll, ΔYaw) between the patient's current body surface and the reference surface. Therapists adjusted the treatment couch or fine‐tuned the patient's posture based on this real‐time feedback until all deviations were within the preset tolerance limits (< 2 mm for translational errors, < 1° for rotational errors).The standardized patient positioning and the real‐time SGRT monitoring interface are illustrated in [Figure 1, 2]

Patient immobilization in the frog‐leg position using a customized vacuum cushion. Note the abduction of the lower limbs to fully expose the inguinal region for radiation delivery. (Note: The modesty sheet shown in the photograph was used for privacy purposes during imaging and was entirely removed from the treatment area prior to SGRT monitoring and radiation delivery to ensure full visibility of the pelvic surface.)

Following the initial alignment phase (either laser‐based or SGRT‐assisted), all patients in both cohorts were subjected to a mandatory daily on‐board helical FBCT scan. This secondary verification served as the clinical gold standard to ensure that all reported “setup errors” accurately reflect the residual deviations of the target volume relative to the initial positioning state, rather than reflecting differences in the final treatment delivery position.

### 2.4 Data acquisition and verification

For all patients in both cohorts, daily image guidance was performed using the on‐board helical FBCT. To ensure target positioning accuracy in the presence of soft‐tissue deformation, a two‐step registration protocol was implemented: (1) Initial skeletal alignment: FBCT images were first rigidly registered to the planning CT based on pelvic bony anatomy and femurs. (2) Manual soft‐tissue fine‐tuning: Subsequently, senior radiation therapists performed manual adjustments to align the soft‐tissue Clinical Target Volume (CTV), focusing on the vulvar lesion and nodal regions. The final recorded shifts reflect this optimized clinical target alignment. The resulting deviation values were recorded as the “setup error” for that fraction.

To prevent statistical bias arising from variations in total treatment fractions, a standardized data truncation method was employed: only the first 25 consecutive FBCT registration datasets were extracted for each patient. Consequently, 250 datasets were obtained per group, totaling 500 independent observations for statistical analysis, as shown in Table 1 and 2.

### 2.5 Statistical Analysis

Data analysis was performed using Python (SciPy library) and SPSS 31.0.

‐ Descriptive Statistics: Mean and Standard Deviation (SD) were calculated for errors in all directions.
‐ Hypothesis Testing: Independent samples t‐tests were used to compare differences between groups.
‐ Effect Size: Cohen's *d* was introduced to quantify the magnitude of differences (standards: *d* ≈ 0.2 small, *d* ≈ 0.5 medium, *d* > 0.8 large effect).
‐ Significance: A two‐tailed *p*‐value < 0.05 was considered statistically significant.
‐ BMI Analysis Strategy: Baseline BMI was recorded (Conventional mean: 24.5 kg/m^2^; SGRT mean: 25.1 kg/m^2^). Considering the sample size (*n* = 10), Spearman's rank correlation analysis was used to evaluate the linear association between BMI and setup errors. 95% Confidence Intervals (95% CI) were reported to quantify uncertainty inherent in small sample studies.

## 3 RESULTS

**FIGURE 3:**
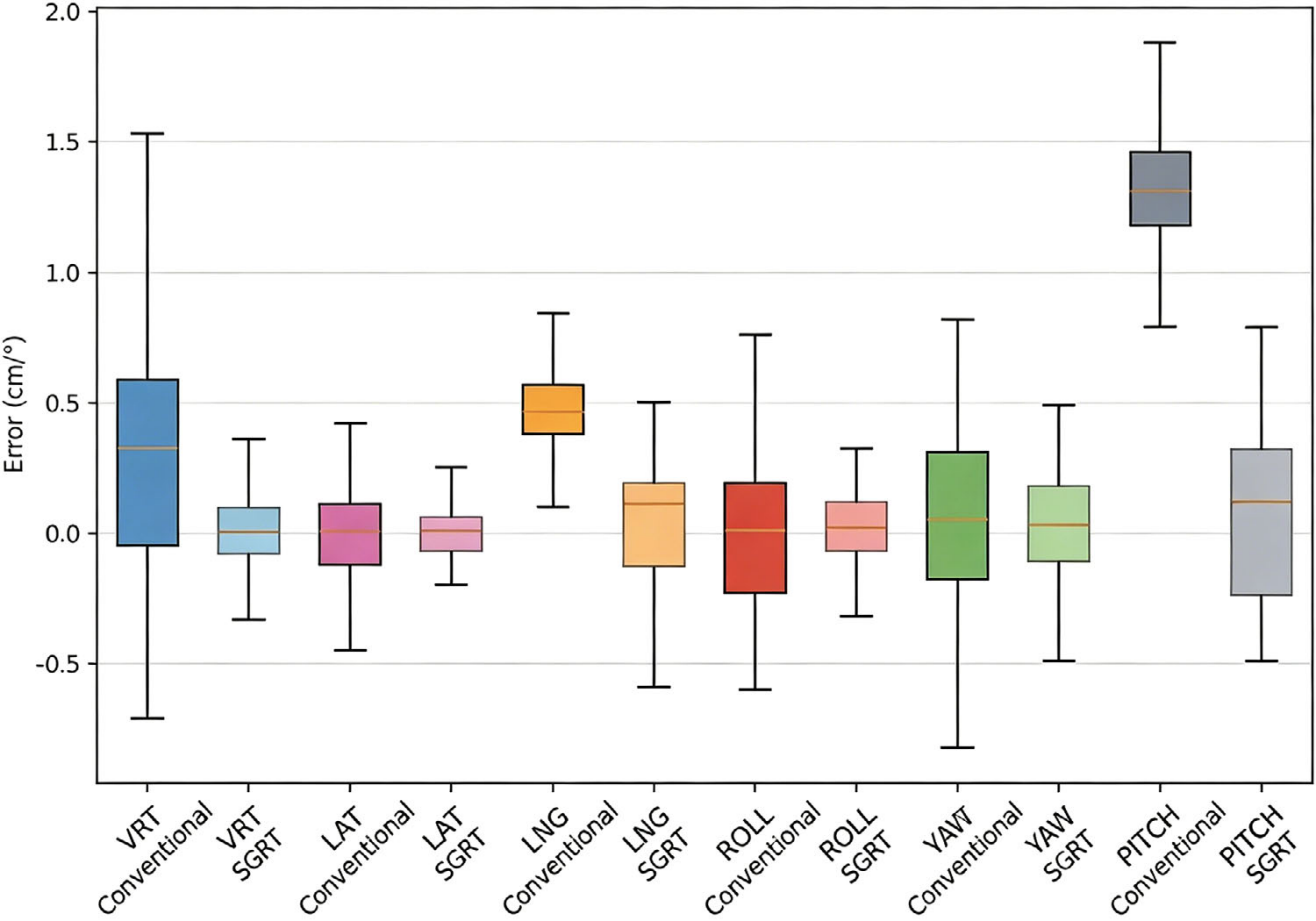
Box plot distribution of setup errors in translational (cm) and rotational (°) directions for Conventional versus SGRT setup methods.

Analysis of the 500 setup verification datasets revealed that the SGRT Group achieved significantly superior setup accuracy across multiple dimensions compared to the Conventional Group (Table 3).[Figure 3] presents box plots comparing the setup errors between the conventional setup and SGRT methods. The results indicate a general trend of improved accuracy and precision with SGRT usage. The SGRT group exhibited narrower interquartile intervals (Q1 to Q3) and median values closer to zero across all six dimensions (VRT, LAT, LNG, ROLL, YAW, and PITCH). In particular, the PITCH direction showed the most substantial reduction in both systematic offset and variance.

**TABLE 1:** Baseline patient characteristics.

Characteristic	Conventional group (*n* = 10)	SGRT group (*n* = 10)
Median Age (years)	58	61
Mean BMI (kg/m^2^)	24.5	25.1
*Clinical Stage (*n*)		
IB	1	1
II	6	7
III	3	2
Radiotherapy Type (*n*)		
Adjuvant	6	6
Definitive	4	4

*Note*: Data are presented as medians, means, or frequencies (*n*) as indicated.

Abbreviations: AJCC, American Joint Committee on Cancer; BMI, body mass index; SGRT, surface‐guided radiotherapy.

^*^Clinical staging was determined according to the AJCC Cancer Staging Manual (8th edition).

**TABLE 2:** Individual BMI data of the two groups.

Patient ID	Conventional Group BMI (kg/m^2^)	SGRT Group BMI (kg/m^2^)
P01	21.2	22.4
P02	22.9	24.1
P03	23.1	28.5
P04	23.5	23.2
P05	24.1	25.8
P06	24.2	26.7
P07	25	24.5
P08	26.4	21.9
P09	26.8	27.3
P10	27.8	26.6

**TABLE 3:** Comparison of setup errors between conventional and SGRT groups.

Variable	Conventional (*N* = 250)	SGRT (*N* = 250)	*t*‐value	*p*‐value	Cohen's *d*	Result
Vertical (cm)	0.285 ± 0.462	0.010 ± 0.150	8.97	< 0.001	0.8	Sig.
Lateral (cm)	0.001 ± 0.163	0.000 ± 0.096	0.07	0.944	0.01	NS
Longitudinal (cm)	0.487 ± 0.256	0.036 ± 0.241	20.3	< 0.001	1.82	Sig.
Roll (°)	0.001 ± 0.301	−0.052 ± 0.312	1.9	0.058	0.17	NS
Yaw (°)	−0.127 ± 0.790	−0.049 ± 0.389	−1.39	0.166	−0.12	NS
Pitch (°)	1.214 ± 0.481	0.069 ± 0.325	31.19	<0.001	2.79	Sig.

Abbreviations: Sig. = Statistically Significant; NS = Not Significant.

### 3.1 Translational errors

Longitudinal (LNG): This dimension showed the most pronounced intergroup difference. The mean deviation in the Conventional Group was 0.49 ± 0.26 cm, whereas the SGRT Group significantly reduced this to 0.04 ± 0.24 cm (*p* < 0.001), with a Cohen's *d* of 1.82, indicating a large effect size.

Vertical (VRT): SGRT precision was significantly better than the Conventional Group (SGRT: 0.01 ± 0.18 cm vs. Conventional: 0.28 ± 0.35 cm, *p* < 0.001).

Lateral (LAT): No significant statistical difference was observed between groups (0.001 ± 0.163 cm for Conventional vs. 0.000 ± 0.096 cm for SGRT, *p* = 0.944), with errors contained within a minimal range in both cohorts.

### 3.2 Rotational errors

Pitch: This was the core finding of the study. The Conventional Group exhibited significant pelvic tilt error (mean 1.21° ± 0.48°). By monitoring abdominal surface slope changes, SGRT reduced this error to 0.07° ± 0.32° (*p* < 0.001), yielding a substantial effect size (Cohen's *d* = 2.79).

Roll and Yaw: No statistically significant differences were found in these dimensions (Roll: Conventional: 0.001 ± 0.301° vs. SGRT: −0.052 ± 0.312°, *p* = 0.058; Yaw: Conventional: −0.127 ± 0.790° vs. SGRT: −0.049 ± 0.389°, *p* = 0.166), with small error magnitudes in both groups.

### 3.3 BMI correlation analysis

Deep mining of the 500 fraction datasets revealed that BMI is a non‐negligible confounding variable in conventional setup.

Conventional Group: BMI showed a strong, significant correlation with Pitch and Yaw errors (*ρ* = −0.81, *P *= 0.005, 95% CI [−0.95, −0.34]), suggesting that higher BMI may exacerbate rotational instability.

SGRT Group: No statistically significant correlation was found between BMI and any setup error dimension (Pitch: *ρ* = 0.25, *P *= 0.489, 95% CI [−0.47, 0.77]). Detailed correlation data are presented in Table 4.

**TABLE 4:** Spearman's rank correlation analysis between BMI and setup errors.

Group	Metric	*ρ* (Coefficient)	*p*‐Value	95% CI	Interpretation
Conventional	VRT	0.53	0.12	[−0.17, 0.87]	Moderate (NS)
	LAT	0.55	0.1	[−0.14, 0.88]	Moderate (NS)
	LNG	0.08	0.83	[−0.59, 0.69]	Negligible (NS)
	ROLL	0.55	0.1	[−0.14, 0.88]	Moderate (NS)
	YAW	−0.81	0.005	[−0.95, ‐0.34]	Strong (Sig.)
	PITCH	−0.81	0.005	[−0.95, ‐0.34]	Strong (Sig.)
SGRT	VRT	−0.1	0.78	[−0.70, 0.58]	Negligible (NS)
	LAT	0.43	0.21	[−0.29, 0.84]	Moderate (NS)
	LNG	0.03	0.93	[−0.62, 0.66]	Negligible (NS)
	ROLL	0.03	0.93	[−0.62, 0.66]	Negligible (NS)
	YAW	0.03	0.93	[−0.62, 0.66]	Negligible (NS)
	PITCH	0.25	0.49	[−0.47, 0.77]	Weak (NS)

Note: Correlation strength criteria: Negligible correlation (∣ρ∣ < 0.2); Weak correlation (0.2≤∣ρ∣ < 0.4); Moderate correlation (0.4≤∣ρ∣ < 0.6); Strong correlation (∣ρ∣≥0.6).

Statistical method: Spearman's rank correlation analysis was used to evaluate the association between BMI and setup errors, with 95% CIs reported to quantify uncertainty in small sample size (*n* = 10).

Abbreviations: SGRT = Surface‐Guided Radiation Therapy; VRT = Vertical; LAT = Lateral; LNG = Longitudinal; ROLL = Roll angle; YAW = Yaw angle; PITCH = Pitch angle; NS = Not Significant (*P* ≥ 0.05).

**FIGURE 4:**
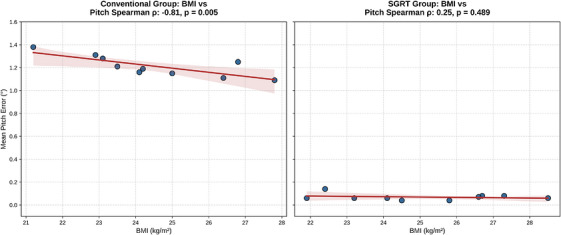
Spearman's rank correlation analysis between BMI and Pitch error for the Conventional Group (Left) and SGRT Group (Right). The red line represents the linear regression fit with the 95% confidence interval (shaded area). A significant correlation is observed only in the Conventional Group (*ρ* = −0.81, *P* = 0.005), whereas SGRT maintains stability independent of BMI.

The divergent impact of BMI on setup stability is visually contrasted in [Figure 4]. As illustrated in the scatter plots, the Conventional Group exhibits a distinct negative linear regression, confirming that higher BMI may be a predictor for increased pitch error magnitude. In contrast, the SGRT Group displays a scattered distribution with a flattened regression line, visually corroborating that surface guidance effectively weakens the correlation between patient BMI and setup errors.

## 4 DISCUSSION

### 4.1 Biomechanical instability in frog‐leg position and SGRT advantages

It is important to clarify that daily image guidance in this study used helical FBCT with a two‐step registration workflow: initial rigid registration to pelvic bony anatomy, followed by manual fine‐tuning of the soft‐tissue target volume. Bony alignment alone does not fully represent soft‐tissue target position in vulvar cancer due to interfractional deformation and frog‐leg position instability. Based on our clinical observations, placing vulvar cancer patients in the frog‐leg position creates a unique pattern of setup errors. Specifically, we noted large deviations in pelvic Pitch and longitudinal displacement.

The biomechanical mechanism can be attributed to the “dynamic equilibrium” of the pelvis when the hip joints are abducted and externally rotated. In this state, minor relaxation or tension in the lumbar muscles can cause the pelvis to tilt anteriorly or posteriorly using the femoral heads as fulcrums. Conventional lateral tattoos are located near this axis of rotation; consequently, pelvic rotation results in minimal displacement of the skin markers, rendering it imperceptible to visual inspection^16^

The core advantage of SGRT lies in replacing “point monitoring” with “surface monitoring.” When pelvic tilt occurs, the topographical gradient of the lower abdomen changes distinctly. SGRT captures these subtle volumetric changes in real‐time, providing visual feedback that allows for correction prior to FBCT acquisition and detection during the beam‐on time of treatment^10^, ^11^ This mechanism is statistically supported by our findings: the reduction of Pitch error from 1.21° ± 0.48° to 0.07° ± 0.32° (*p* < 0.001), with a massive effect size (*d* = 2.79), confirms the decisive role of SGRT in correcting pelvic tilt. These findings align with recent data reported by Bolin et al.^17^

### 4.2 Correction of longitudinal errors

We attribute the ∼5 mm longitudinal error in the Conventional Group to a “skin traction effect.” When patients open their legs into the immobilization cushion, the skin of the lower abdomen and groin pulls downward. While lasers might still line up with these stretched skin marks, the actual internal target shifts cranially. Because SGRT tracks the patient's actual 3D surface instead of ink marks, it naturally avoids these skin‐sliding errors^14^ In our study, this approach brought the longitudinal error down to just 0.04 ± 0.24 cm (*p* < 0.001) in the SGRT group. The large effect size (*d* = 1.82) shows that SGRT nearly eliminates systematic bias in this direction.

### 4.3 Clinical implications and dosimetric impact

High‐precision setup translates directly into dosimetric benefits, with Pitch error being particularly critical in pelvic radiotherapy. Previous studies indicate that rotational setup errors, particularly a pelvic tilt (pitch) exceeding 1°, can be magnified in elongated pelvic targets^16^ Such deviations may cause the presacral lymph node target to shift posteriorly out of the planned high‐dose region, or inadvertently displace the small bowel into the high‐dose field, thereby increasing the risk of gastrointestinal toxicity. In our study, SGRT controlled Pitch error to within 0.1°. This implies that clinicians can optimize Planning Target Volume (PTV) margins with greater confidence, potentially reducing the traditional 5–7 mm margin to 3–5 mm^18^ This would further decrease the dose delivered to the rectum, bladder, and femoral heads. Additionally, reducing the need for large FBCT shifts enhances treatment efficiency and minimizes additional radiation exposure from repeated scans.

Furthermore, the translation of geometric precision into dosimetric advantage is particularly critical for the unique anatomy of vulvar cancer. In the frog‐leg position, the inguinal lymph nodes are situated superficially, yet in close proximity to critical structures such as the femoral heads and the small bowel. A pelvic pitch error exceeding 1° can significantly alter the radiological depth of the nodes relative to the incident beam, potentially compromising target coverage or introducing hot spots in the skin folds. By constraining rotational errors to a sub‐degree level (< 0.1°), SGRT effectively minimizes the “blurring” of steep dose gradients. This geometric confidence provides a radiobiological rationale for safely reducing the PTV margin from the conventional 5–7 mm to a tighter 3–5 mm range.

The significant reduction in Longitudinal and Pitch errors observed in the SGRT group is highly consistent with the latest international evidence. A recent systematic review and meta‐analysis by Rudat et al. (2023)^18^ synthesized data from multiple clinical trials, providing robust confirmation that SGRT significantly reduces both systematic and random setup errors compared to laser‐based setup. Our results align with the trends observed in Rudat ’s large‐sample analysis, corroborating the reliability of our data. However, a critical distinction lies in the patient positioning; the aforementioned meta‐analysis predominantly included studies utilizing the standard supine position. In contrast, our study focuses on the frog‐leg position, which presents a more complex biomechanical structure. Unlike the supine position, the abduction of the lower limbs in the frog‐leg position induces a significant “skin traction effect,” particularly in patients with high BMI, which leads to the potential failure of traditional laser localization. Our data provide the first quantitative assessment of how SGRT effectively eliminates BMI interference with pelvic Pitch in this specific position. This not only demonstrates that the high‐precision advantages of SGRT are applicable to the biomechanically unstable frog‐leg position but also fills a critical gap in evidence‐based medicine for optimizing PTV margins in this specific patient population.

While daily FBCT theoretically provides the definitive positional correction, our findings underscore a critical synergy between SGRT and internal imaging that extends beyond simple translation shifts. Crucially, the clinical targets in vulvar malignancy consist primarily of superficial soft tissues, which exhibit more pronounced interfractional morphological variation than osseous landmarks or deep‐seated tumors. SGRT facilitates superior alignment of these soft‐tissue contours by providing a global topographical match rather than relying on localized skin markers.

This surface‐matching capability is particularly vital for delivery systems without a six‐degree‐of‐freedom (6‐DoF) robotic couch, such as the uRT‐linac 506c used in this study. On such platforms, rotational offsets—specifically pelvic Pitch—cannot be corrected via automated couch movements. By identifying and allowing for manual correction of these rotations during the initial setup, SGRT ensures that the soft‐tissue targets are aligned with maximum conformality before the beam‐on time. This proactive approach not only mitigates the risk of geographical miss but also significantly improves workflow efficiency by reducing the necessity for repeated FBCT scans and repositioning due to out‐of‐tolerance rotations.

### 4.4 BMI correlation

In the frog‐leg position, patients with higher BMI may present with thicker adipose layers in the abdominal and inguinal regions, leading to more pronounced skin‐to‐bone displacement. Traditional setup markers are vulnerable to this “skin traction effect,” making it difficult for therapists to visually identify deep pelvic rotational errors. Our findings resonate with those of Baba et al.^19^ and Haslam et al.^10^ who concluded that high BMI is potentially associated with larger setup errors in pelvic radiotherapy and reduced reliability of skin marks. The core advantage of SGRT lies in its unique “surface matching” mechanism: even in high BMI patients, the technology accurately corrects Pitch errors by capturing global changes in lower abdominal topology. This robustness ensures consistent high‐precision radiotherapy across varying BMI levels, providing a strong rationale for reducing unnecessary PTV margins in obese patients.

### 4.5 Limitations

This study has several limitations:(1) The sample size was relatively small (*n* = 20), although 500 observational datasets were included to enhance statistical power; further validation in larger multi‐center cohorts is warranted.(2) Although we used a two‐step FBCT registration (bony anatomy + soft‐tissue fine‐tuning), bony anatomy remained the primary initial reference. Vulvar cancer targets are soft‐tissue structures that undergo significant interfractional deformation; thus, reported setup accuracy mainly reflects skeletal alignment precision and may not fully represent true soft‐tissue target positioning. Accordingly, inferences regarding PTV margin reduction should be interpreted with this constraint.(3) This study focused on geometric setup errors and did not directly evaluate dosimetric differences or clinical outcomes (e.g., local control or skin toxicity), which require longer‐term follow‐up.

## 5 CONCLUSION

In conclusion, for patients with vulvar cancer treated in the frog‐leg position, traditional laser‐skin marking methods may fail to effectively control pelvic tilt (Pitch) and longitudinal sliding errors. SGRT, as a non‐ionizing, real‐time, six‐dimensional guidance modality, significantly reduces these specific setup errors, offering precision superior to conventional methods. Given the inherent biomechanical instability of the frog‐leg position, our study demonstrates that routine integration of SGRT in the treatment of vulvar cancer may improve both setup stability and accuracy before and throughout treatment.

## AUTHOR CONTRIBUTIONS

**Hao Liang**: Methodology; data curation; formal analysis; writing—original draft. **Junfang Yan**: Resources; validation. **Yongguang Liang**: Investigation; software. **Xiansong Sun**: Conceptualization; writing—review & editing; project administration. **Hongming Li**: Supervision. **Huiying Qu**: Visualization. **Yijun Wang**: Data collection. **Jingyu Lin**: Statistical analysis; manuscript revision.

## CONFLICT OF INTEREST STATEMENT

The authors declare that they have no known competing financial interests or personal relationships that could have appeared to influence the work reported in this paper.

## ETHICS APPROVAL

This study was approved by the Ethics Committee of Peking Union Medical College Hospital (Ethics No. JS‐3530).

## Supporting information




Supporting Information

## Data Availability

The datasets generated and/or analyzed during the current study are available from the corresponding authors on reasonable request.
