# Supplementary material for: Surface‐Guided Radiotherapy for Vulvar Cancer in the Frog‐Leg Position: Setup Accuracy and BMI Effects
Source: J Appl Clin Med Phys. 2026 Jul 26;27(8):e70714. doi: 10.1002/acm2.70714 (PMC13402252; doi:10.1002/acm2.70714)
Supplement: Supplementary file 1 — Supporting Information [file ACM2-27-e70714-s001.docx]

**itle:** Surface-Guided Radiotherapy (SGRT) in Vulvar Cancer Patients Treated in the Frog-Leg Position: A Clinical Study on Setup Accuracy and the Biomechanical Impact of BMI

**Running Title:** SGRT for Vulvar Cancer in Frog-Leg Position

**Authors:** Hao Liang<sup>1</sup>, Junfang Yan<sup>1</sup>, Yongguang Liang<sup>1</sup>, Xiansong Sun<sup>1, *</sup>, Hongming Li<sup>1</sup>, Huiying Qu<sup>1</sup>, Yijun Wang<sup>1</sup>, and Jingyu Lin<sup>1</sup>

**Affiliations:** <sup>1</sup> Department of Radiation Oncology, Peking Union Medical College Hospital, Chinese Academy of Medical Sciences and Peking Union Medical College, Beijing, China

**Corresponding Author:**

**Xiansong Sun** Department of Radiation Oncology, Peking Union Medical College Hospital No. 1 Shuaifuyuan, Dongcheng District, Beijing 100730, P.R. China E-mail: sxs231@163.com

**Conflict of Interest:** The authors declare that they have no known competing financial interests or personal relationships that could have appeared to influence the work reported in this paper.

**Funding:** This research received no specific grant from any funding agency in the public, commercial, or not-for-profit sectors.

**Data Availability Statement:** The datasets generated and/or analyzed during the current study are available from the corresponding authors on reasonable request.

**Ethics Approval:** This study was approved by the Ethics Committee of Peking Union Medical College Hospital (Ethics No. JS-3530).

**Keywords:** Vulvar Neoplasms; Frog-leg Position; Surface-Guided Radiotherapy (SGRT); Setup Errors; Body Mass Index (BMI)
